# Common Neuroanatomical Substrate of Cholinergic Pathways and Language-Related Brain Regions as an Explanatory Framework for Evaluating the Efficacy of Cholinergic Pharmacotherapy in Post-Stroke Aphasia: A Review

**DOI:** 10.3390/brainsci12101273

**Published:** 2022-09-21

**Authors:** Marina Katsari, Georgia Angelopoulou, Nikolaos Laskaris, Constantin Potagas, Dimitrios Kasselimis

**Affiliations:** 1 Neuropsychology and Language Disorders Unit, 1st Department of Neurology, Eginitio Hospital, National and Kapodistrian University of Athens, 115 28 Athens, Greece; 2 Department of Speech and Language Therapy, School of Health Sciences, University of Peloponnese, 241 00 Kalamata, Greece; 3 Department of Industrial Design and Production Engineering, School of Engineering, University of West Attica, 122 43 Athens, Greece; 4 Department of Psychology, Panteion University of Social and Political Sciences, 176 71 Athens, Greece

**Keywords:** post-stroke aphasia, recovery, pharmacotherapy, acetylcholine, donepezil

## Abstract

Despite the relative scarcity of studies focusing on pharmacotherapy in aphasia, there is evidence in the literature indicating that remediation of language disorders via pharmaceutical agents could be a promising aphasia treatment option. Among the various agents used to treat chronic aphasic deficits, cholinergic drugs have provided meaningful results. In the current review, we focused on published reports investigating the impact of acetylcholine on language and other cognitive disturbances. It has been suggested that acetylcholine plays an important role in neuroplasticity and is related to several aspects of cognition, such as memory and attention. Moreover, cholinergic input is diffused to a wide network of cortical areas, which have been associated with language sub-processes. This could be a possible explanation for the positive reported outcomes of cholinergic drugs in aphasia recovery, and specifically in distinct language processes, such as naming and comprehension, as well as overall communication competence. However, evidence with regard to functional alterations in specific brain areas after pharmacotherapy is rather limited. Finally, despite the positive results derived from the relevant studies, cholinergic pharmacotherapy treatment in post-stroke aphasia has not been widely implemented. The present review aims to provide an overview of the existing literature in the common neuroanatomical substrate of cholinergic pathways and language related brain areas as a framework for interpreting the efficacy of cholinergic pharmacotherapy interventions in post-stroke aphasia, following an integrated approach by converging evidence from neuroanatomy, neurophysiology, and neuropsychology.

## 1. Background

Aphasia is rather common among left brain damaged stroke survivors. Its prevalence among stroke patients can be as high as 38% [1], while according to current evidence, 38% of stroke survivors may be suffering from aphasia in the USA [2]. The impact of post-stroke aphasia is not restricted to language deficits. There is increasing evidence that aphasic disturbances often coexist and correlate with deficits in other aspects of cognition see for example: [3,4,5]. Moreover, aphasia has a significant impact on psychosocial aspects of the patients’ life, since they impose severe limits to everyday living activities, thus reducing quality of life [2]; aphasia has been importantly associated with several psychological issues and mostly post-stroke depression for two recent systematic reviews, see: [6,7].

There is a significant number of studies supporting the hypothesis that spontaneous recovery of language and other cognitive functions is a dynamic process that mostly occurs during the first six months after stroke; it involves the functional reorganization of a broaden neural network beyond traditional language-related areas within perisylvian network of the language dominant hemisphere but also the homologue regions of the contralateral hemisphere see for a review: [8]. During the first days after stroke (acute phase), an activation in intact left-hemispheric (language-related) areas is revealed, which is gradually expanded within the perisylvian network but also domain-general areas bilaterally, with a peak observed in right lesion-homologue regions including Broca’s and supplementary motor area (SMA). Finally, as patients progress to the chronic phase, a normalization of activation patterns in the left hemisphere is gradually established. These patterns seem to be related with recovery of core language functions, as spontaneous speech output, comprehension, and repetition, in both acute and chronic phases [9,10,11]. However, it should be noted that more recent evidence suggest that language reorganization is highly dependent on specific lesion loci, thus different activation patterns may appear in the lesion-homologue brain areas of the right hemisphere [12], in cases of frontal and posterior lesions [13].

Aphasia rehabilitation via traditional speech and language therapy (SLT) has been shown to be beneficial as it usually focuses on the exact nature of individual language deficits (impairment-based therapy), but its value is time-restricted, in the sense that its positive impact is limited especially for patients in the chronic stage. Improvement of language skills can be observed in chronic aphasic patients after SLT [14,15,16,17], nevertheless, the vast amount of recovery is more or less completed 6–12 months post onset [18,19], i.e., the clinical image of the patient is stable at that time, with possible minor improvement [1]. Even in cases of intensive therapeutic programs during the chronic phase of stroke, benefits may appear to be relatively limited in several cases of patients: see [20], for a recent systematic review. More importantly, despite the effectiveness of impairment-driven therapies, it seems that for a significant number of stroke survivors, especially those with severe language deficits, observed recovery is limited [21]. Even for the more recent “functional based” therapeutic approaches, in which specialists center their attention in linguistic and extra-linguistic communication skills, results with regard to their effectiveness are relative scarce and not well-established [22]. So far, there is a limited number of studies providing comparative evidence with regard to the impact of different types of SLT in the same individuals [23]. More studies are necessary in order to evaluate the effectiveness of various SLT approaches, in post-stroke patients with specific language deficits profiles, in order to clarify the impact of certain therapeutic programs during the course of spontaneous recovery and, more importantly, to further elaborate on specific strategies for the number of patients that continue to confront language difficulties, after the completion of intervention programs.

In sum, despite the positive impact of SLT programs, there are limited options for a significant number of patients in the chronic stage, with regard to speech therapy methods. It should be also noted that there are also other factors that may affect SLT efficacy, such as increased health-care costs [24,25] or other environmental dimensions, including healthy and social crises (for instance: COVID-19 pandemic [26]). The consequences of such conditions may be patients with restricted access to SLT therapy [24], as well as alternative therapeutic approaches, such as telerehabilitation, which, despite limited evidence, seems promising [27,28,29,30]. Therefore, other types of intervention as pharmacotherapy would be an additional, possibly beneficial, choice for individuals who suffer from chronic aphasic symptoms. Especially when the lesion affects subcortical regions, and part of the language cortex remains intact, aphasic symptoms could be attributed to disruption within a cortical–basal ganglia–thalamic circuit see for example: [31,32,33] or interruption of ascending neurotransmitter systems [34], in which case a pharmacological response might be expected. 

Studies on pharmacological remediation of acquired language disorders are characterized by great heterogeneity, involving small numbers of patients and varying types of aphasia [11,35]. In addition, it is frequently difficult to differentiate between improvement due to spontaneous recovery and recovery due to treatment. However, some of these studies gave promising results in the sense that in some cases, the language deficits of patients with aphasia can be improved with pharmacotherapy. Indeed, an extensive review of the literature reveals that pharmacotherapy can be beneficial with regard to post-stroke aphasia’s outcome [1,22,23,35,36,37,38,39,40,41,42,43], yet results in some cases appear still controversial [see for a critical discussion 11]. Several dopaminergic, cholinergic, noradrenergic, and glutamatergic agents have been used in this field [23,38,41,42,43,44,45]. All in all, existing evidence in post-stroke aphasia pharmacotherapy indicates beneficial outcome in a wide range of speech, articulation and language sub-processes such as naming and comprehension skills [23,38]. It is therefore suggested that pharmaceutical augmentation could be an advantageous choice for those individuals who suffer from chronic aphasic symptoms, based on the hypothesis that neurotransmitters’ modulation may enhance reorganization of brain-related areas [23].

According to existing literature, catecholamines seem promising for patients with post-stroke aphasia, as the decreased level of cerebral catecholamines induced by cerebral infarction has been suggested to play an important role in impaired function, including aphasia. So far, the majority of studies seem to focus their interest on the investigation of catecholamines’ effects in post-stroke aphasia recovery [38]. Nevertheless, there is significant evidence that increase of acetylcholine concentrations seems also to improve language disorders’ symptomatology in patients with post-stroke aphasia [11,46,47,48]. In the current review, we are going to focus on the effects of cholinergic pharmacotherapy on different aspects of language deficits post-stroke. In particular we will attempt to set an explanatory framework of the efficacy of such therapies, on the basis of the common neuroanatomical substrate of cholinergic pathways and language-related brain areas.

## 2. Acetylcholine, Cognition, and Plasticity

Extensive evidence (ranging from experiments assessing the effects of loss of cortical cholinergic inputs on human cognition to studies assessing cortical acetylcholine release in task performing animals), has substantiated the general hypothesis that cortical cholinergic inputs primarily mediate attention process and capacities [49]. Limitations with regard to attention and available processing resources are related with reduced encoding efficiency [50,51] deficient rehearsal, and overall decreased memory capacity [52].

A great proportion of presynaptic cholinergic receptors in the brain consist of nicotinic receptors [53]. Due to their location, they are involved in other neurotransmitter systems, thus regulating neuromodulatory networks that are essential for cognitive functions [48].

Acetylcholine (Ach) is suggested to play an important role in neuroplasticity [54,55]. It has been observed in both animal models and studies in humans that agonists of nicotinic receptors (nAChRs) have a long-lasting outcome in cognition. More importantly, it has been shown that the duration of these cognitive effects may survive for a longer period of time than the duration of the agonists’ presence in the brain, while the tenacity of cognitive enhancement may be further increased with repetitive exposure. Agonists of nAChRs induce long-term potentiation (LTP), which is associated with learning and memory. Some of the effects of nAChR agonists at the cellular level overlap with the known cellular mechanisms of LTP, including long-lasting increases in intracellular concentrations of Ca^2+^, activation of second-messenger systems and transcription factors, enhanced gene expression, and increased release of neurotransmitters. A better understanding of this phenomenon might shed new light on the role of nAChR systems in memory formation and retrieval [56,57].

Finally, it has been shown that nerve growth factor (NGF) and brain-derived neurotrofin factor (BDNF) mRNA and NGF protein are up-regulated in the rat hippocampus by the activation of muscarinic receptors. However, evidence from studies in rats indicated that NGF and BDNF may stimulate ACh release from hippocampal synaptosomes which include the terminal buttons of septal cholinergic neurons. NGF also rapidly increases the high-affinity choline transport into synaptosomes. The reciprocal regulation of ACh, NGF and BDNF in the hippocampus influence synaptic plasticity [58,59].

In sum, cholinergic cortical projections seem to be anatomically/pharmacologically optimal to modulate neocortical plasticity, with respect to acetylcholine neuromodulation. This system has an important role in attention, and memory: stimuli that are arousing and of attentive value are those that should induce plastic changes in the brain.

## 3. Neural Substrate of Language and Related Cognitive Domains 

Contemporary models suggest the existence of complex brain networks related to core language functions as phonological and semantic processing, while the so-called “domain-general” brain areas also seem to have a significant role in language. Saur and colleagues [59] empirically assessed the theoretical dual stream model for language introduced by Hickok and Pοeppel [60]. They found that phonological processing seems to be supported by a dorsal stream of cortical and subcortical areas including the pars opercularis (BA 44) and the premotor area (BA 6) connected via the arcuate fasciculus and the third branch of superior longitudinal fasciculus with the posterior part of the inferior parietal lobule and the superior temporal gyrus [61]. Semantic processing, on the other hand, is related to a ventral stream which includes the pars triangularis (ΒA 45) and pars orbitalis (ΒA 47) linked with posterior temporal regions (mainly the middle temporal gyrus) via the temporofrontal extreme capsule fasciculus [62]. Additional evidence, in favor of the existence of such connections derives from comparative studies in other primates, such as macaque monkeys; see for example: [63,64]. Moreover, Indefrey and Levelt [65] in a metanalysis of eighty-two fMRI studies identified brain areas which appear to be critical for the various steps of single word production, including, beyond traditionally language-related areas, the supplementary motor areas, the insula and the thalamus. The role of the thalamus and the insula has been well-established in language sub-processes [46,66,67,68,69]. Posterior left ventrolateral and pulvinar thalamic lesions have been shown to result in severe word-finding deficits, along with mild disturbances in reading, repetition and auditory comprehension [70]. Similar evidence is also derived from stimulation studies [70]. Dronkers [71] found that stroke patients with insular lesions have severe deficits in planning articulation. Lesion studies also suggest that the insula may be related with processing of phonological [3,72,73,74,75] but also semantic information [76], while it is also assumed to play a significant role in selective retrieval of verbal information from temporal cortices, along with temporofrontal Extreme Capsule Fasciculus [77].

As will be discussed in the following section, several cortical areas, which have been related to different language functions, receive cholinergic input via two major distinct bundles of white matter fibers, the medial and the lateral pathway. Especially, the lateral pathway in its capsular division seems to include neighbor white matter tracts, such as the external capsule and the uncinate fasciculus [78,79], which are considered part of the ventral pathway for language semantic processing [59,62], while the perisylvian division includes fontoparietal and temporal areas [79]. Thus, we could claim that since association cortices and white matter tracts previously related with language functions, receive cholinergic input, this consists of an additional argument that acetylcholine may have a significant role in language processing, beyond other aspects of cognition as memory and attention. On the other hand, we should consider that language shares a common neural substrate with other cognitive functions [80]. For instance, lesion and functional brain imaging studies reveal an overlap, and possibly a common substrate between language processing and working memory. More specifically, lesion studies suggest a relation between posterior regions, such as the superior temporal gyrus, and working memory [80,81]. Modern lesion studies confirm the traditional notion that the left posterior temporo-parietal region is crucial for comprehension, pointing out that posterior lesions, including the superior and middle temporal gyrus, and the inferior parietal lobule, are related to comprehension impairment in aphasia [82,83]. According to functional brain imaging studies increased activation of prefrontal and parietal areas is related to verbal working memory tasks [84,85]. These areas have also been involved in lexical-semantic processing [86,87]. The above data present a clear-cut overlap with regard to the neural substrate of language and working memory, therefore it could be hypothesized that both functions are parallel processes in terms of anatomy and physiology. Finally, we should not ignore the fact that working memory deficits have been reported in several studies in post-stroke aphasia [5,81,88,89,90,91], which, in some cases, are not restricted to verbal modality [4,5,89]. Brain imaging studies confirm such clinical data by demonstrating the involvement of the left hemisphere in non-verbal stimulus processing [91]. Caplan [92] has suggested that some aspects of language impairment may be related to such deficits, in the sense that comprehension deficits in aphasia are due to resource reduction. In sum, converging evidence suggests that the language functions are interwoven with other aspects of cognition both in terms of their neurobiological substrate and observable behavior.

To conclude, cholinergic pathways seem to support networks of brain areas related with different aspects of cognition, such as attention, memory but also language, which are highly depended on the integrity of cholinergic inputs [52]. Thus, it could be hypothesized that these inputs may support the recovery of such functions in case of disruption due to a focal damage [47].

## 4. Cholinergic Pathways in Basal Forebrain-and Perisylvian Language-Related Regions

Study of human but other primates’ brain has indicated that cholinergic input is diffused to whole brain cortical areas, originated by nucleus basalis of Meynert of the basal forebrain [78,93,94,95]. Eight major cholinergic cell groups are considered to project to several brain regions. Ch1 is associated with medial septal nucleus, Ch2 is associated with the vertical nucleus of the Diagonal Band of Broca, Ch3 is associated with the horizontal limp of diagonal band of Broca, Ch4 is associated with the nucleus basalis of Meynert, Ch5 is associated with Penduculopontine Nucleus of the rostral brainstem, Ch6 is associated with the laterodorsal Tegmental Nucleus, also in the rostral brainstem, Ch7 is associated with the medial Habenula, and ch8 with the Parabigeminal Nucleus [78,95,96]. The Ch1–Ch4 groups are the only neurons which regularly express large amounts of NGF receptor in the adult human central nervous system [78].

Of all cholinergic cell groups, the Ch4 group is by far the largest and the one that has been most extensively studied in the human brain [95]. The constituent neurons of the human NB –Ch4 complex can be subdivided into six sectors: The anterior sector (Ch a), which is further divided by vasculature into the anteromedial (Ch4 am) and the anterolateral (ch4al) sectors, the anterointermediate sector (Ch4 ai), which spans the anterior and intermediate sectors, the intermediate sector (Ch4i) which is divided by the Ansa peduncularis into the intermediodorsal (Ch4id) and intermedioventral (Ch4 iv) sectors. The posterior part occupies a sector designated as Ch4p [78,96].

Axonal transport experiments combined with AchE histochemistry in the monkey have shown that Ch1 and Ch2 provide the major source of cholinergic innervation for the hippocampal complex, that Ch3 provides the major source of cholinergic innervations for the olfactory bulb, and that Ch4 is the major source of cholinergic projection for the entire cerebral cortex and the Amygdala.

Different divisions of the Nucleus Basalis have physiologically and morphologically heterogeneous neurons with discrete projectional patterns, indicating that the Nucleus Basalis, is composed of different organizational units. One cell group, which is topographically related to the Nucleus Basalis, and has been classified as the most lateral component of the magnocellular basal forebrain complex, is Nucleus SubPutaminalis (NSP).

The anterointermediate and intermediate levels of the NSP project two fiber bundles ascending together through External Capsule, along the lateral margin of the putamen and separating above its dorsolateral edge, innervating different regions of the cortex: A bundle with medial direction projects to the medial part of the hemisphere (cingulum), while the other one turning around the insular gyrus continue towards the inferior frontal gyrus, which plays a crucial role for language, based on studies in both healthy individuals [97,98,99,100] and patients with acquired language deficits [101].

Knowing that language is unique to humans and that the cholinergic input to the cerebral cortex has a modulatory role for a wide range of cortical functions, particularly those related to memory, learning and attention [99,102,103], it is hypothesized that NSP may provide modulation of these functions in the cortical areas involved in language processing. Studies on dementia provide clues in favor of this view, by showing that degenerative disorders characterized by cholinergic and NGF receptor deficits of the basal forebrain neurons [98], particularly Alzheimer’s Disease, are frequently accompanied by language disturbances [98,103,104,105,106].

## 5. Cholinergic Treatment in Aphasia

Luria and colleagues [107] presented early evidence claiming that galantamine, a cholinesterase inhibitor, may enhance recovery of several language functions such as naming, along with semantic and phonemic information processing. Despite the fact that in most of the studies researchers use catecholaminergic drugs [108], the value of cholinergic drugs, traditionally considered to have a positive impact in memory, learning and attention, is well-established in post-stroke aphasia [23,36,37,38,39,108,109] (see Table 1 for studies using cholinergic treatment in post-stroke aphasia). It has been even argued that cholinergic agents may be more promising in aiding post-stroke aphasia recovery compared to catecholamines [109]. Positive effects in naming recovery have also been reported after administration of cholinergic drugs such as physostigmine [110], bifemelane [111] and ameridin [42]. Nevertheless, most of these agents have not been further tested as bifemelane is available only in Japan while physostigmine is considered as a safe option [38,109]. In most of the studies donepezil is administrated as cholinergic treatment in post-stroke aphasia, while only very few studies have reported results for galantamine [112].

### 5.1. Donepezil

#### 5.1.1. Acute versus Chronic Phase of Post-Stroke Aphasia

Donepezil is the most common cholinergic agent used for the treatment of post-stroke aphasia [36,37,38,39,45,109,121]. During the last twenty years several studies have indicated the beneficial effects of donepezil in patients with post-stroke aphasia, either in case [36,113,118] or group studies, mostly in chronic phase of stroke [114,115,116,119], while sparse evidence exists for the acute phase [117]. In most studies patients were assessed at least one-year post-stroke and no more than four years post-stroke see for example: [113,114,115]. However, there is one study focusing on a patient who was at a later stage (i.e., increased time post-stroke), which provided promising findings. Yoon and colleagues [118] presented a case of a female patient (53 years old) with fluent aphasia, assessed 8 years post stroke. The patient presented severe comprehension deficits and based on positron emission tomographic images, decreased cerebral metabolism in the left temporoparietal area and the right temporal lobe (the latter possibly being due to a subsequent infarct in the right temporal lobe 4 months prior to assessment) prior to treatment. After 12 weeks of Donepezil treatment, the patient exhibited increased metabolic activity in both left and right middle temporal gyri, while comprehension ability was also improved. Despite the fact that the above findings derive from the investigation of only one patient, this study elevates the expectation that pharmacotherapy may enhance recovery in patients with aphasia many years after their cerebrovascular accident, especially when one takes into consideration that traditional speech and language therapy is usually terminated after the first two years post-stroke.

In sum, 110 patients have been investigated in a total number of nine studies, with only four of them being group studies (see Table 1 for a detailed presentation). The range of donepezil treatment duration was between 6 and 25 weeks, and in most of the studies the dose was initially 5 mg, and then was increased to 10 mg. In 6 studies’ research design (3 out of which were group studies), a comparative condition of traditional SLT was included. However, given that each research group implemented a different SLT program, it is rather difficult to comparatively evaluate the effect of pharmacotherapy and traditional SLT therapeutic approaches.

#### 5.1.2. Positive Effects on Language and Other Cognitive Domains

In most studies researchers examined core aspects of language functions to investigate possible gains after administering donepezil. Assessment was mostly accomplished using aphasia batteries and more specifically Western Aphasia Battery (see for example: [36,117,118,119,120,122]). In most of them, Aphasia Battery Quotient, a measure of aphasia severity was considered as a core metric to quantify any change, while the major language domains assessed were spontaneous speech, comprehension, repetition, and naming functions. In some cases, PALPA (Psycholinguistic Assessments of Language Processing in Aphasia) and CAL (Communicative Activity Log), a scale that assesses the patient’s communicative behavior were also used [116]. While results vary, it seems that donepezil could be a good therapeutic option for word-finding and naming deficits [38].

It is noteworthy that very few studies have directly investigated further cognitive functions as verbal short-term/working memory. Berthier and colleagues [117] examined the effect of donepezil along with massed sentence repetition therapy in three patients with chronic post-stroke aphasia due to extensive lesions. Patients were assessed with various tasks of words and sentence repletion and digit span, along with WAB. Results revealed that patients presented improved performance in several repetition tasks and aphasia severity index, while donepezil was more effective when combined with more-intensive therapy for a longer period of time. Woodhead and colleagues [119] also reported the effect of specific phonological training via a software in combination with pharmacological intervention using donepezil. Patients presented improved performance in language comprehension. Sparse evidence also come from studies in children. Dávila and colleagues [122] reported the case of a nine-year-old girl with word-finding difficulties, due to a severe closed TBI. A combined treatment of donepezil and intensive naming therapy had a positive effect on speech output, auditory comprehension, repetition, and picture naming, but also others cognitive functions, such as processing speed and attention. It should be noted that effects of donepezil in child aphasia are rarely tested, and although that study was a case report, it undoubtably offers encouraging results for further research.

## 6. Lesion Site, Pharmacotherapy, and Synaptic Gain

Functional reorganization of spared tissue in left hemisphere after stroke is rarely reported in pharmacotherapy studies. Yoon and colleagues [118] reported increased F-18 FDG uptake in both middle temporal gyri along with improved performance in comprehension, after 3 months of pharmacological intervention with donepezil in a patient with fluent aphasia. Woodhead and colleagues [119] collected EEG and MEG data to investigate effective connectivity and possible synaptic gain in two patients with posterior lesions and severe impairment in comprehension, after administration of donepezil treatment. Results revealed that improvement in auditory comprehension after pharmacotherapy and phonological training was significantly associated with stronger modulation of the left superior temporal gyrus. Especially for patients with severe comprehension impairment, behavioral therapy indicated stronger phonemic sensitivity in the Superior Temporal Gyrus interhemispheric connections, whereas donepezil showed no effect. Limited data also exist for structural changes during post-stroke aphasia recovery. Berthier and colleagues [120] reported that donepezil in combination with audiovisual repetition-imitation therapy led to structural alterations in the right frontal aslant tract and direct segment of the arcuate fasciculus in a 46-year-old patient with global post-stroke aphasia due to a right striatal-capsular haemorrhage. In addition, significant improvement in several aspects of language and communication abilities, such as naming, connected speech and repetition, were observed. However, it should be noted that current results derive from a small sample size and further research is required to elucidate possible functional changes in intact brain areas due to cholinergic treatment and further elaborate on the common neuroanatomical substrate of cholinergic pathways and language related brain areas, to promote cholinergic treatment in post-stroke aphasia.

## 7. Conclusions

In sum, little evidence has been generally reported during the last two decades with regard to aphasia pharmacotherapy and especially the positive effects of cholinergic augmentation. In addition, there are a few neuroimaging studies, which also seem to be in accordance with the hypothesis that cholinergic drugs may be effective for treating aphasic deficits post stroke. This notion is further supported by the fact that specific cortical and subcortical regions that are known to play a significant role in aphasia recovery seem to overlap with neural networks heavily dependent on cholinergic synaptosomes. It could be argued that pharmacotherapy, combined with an effective therapeutic speech and language intervention program could enhance functional reorganization and remediation of language and accompanying cognitive deficits in patients with post-stroke aphasia. Having said that, there are some issues which have to be taken into consideration, such as the fact that some studies raise the concern of side effects and report possible conflicts of interest. Overall, data on cholinergic pharmacotherapy in acquired language disorders may be scarce, but promising. That is why further research is crucial to shed light on this issue, thus contributing to the quest of finding more effective strategies to support individuals with aphasia and promote their quality of daily living.

That being said, we would like to explicitly state that we are far from suggesting that pharmacotherapy should replace traditional SLT therapy in aphasia, nor do we argue that it should be the primary treatment. As already discussed in this paper, SLT’s value has been supported by several papers over the last decades, while the efficacy of pharmacotherapy has been investigated by a relatively scarce number of studies. The main aim of the present review is to set an interpretational framework for the effectiveness of cholinergic agents in aphasia, by integrating evidence from neuroanatomy, neurophysiology, and neuropsychology, and further attempting to investigate possible commonalities between cholinergic networks and widely distributed brain regions, which are known to be associated with language, but also other aspects of cognition that are assumed to be functionally and anatomically interwoven with language, such as the subprocesses of working memory. Thus, working towards understanding the underlying neural mechanisms of pharmacological augmentation in relation to the neurobiology of language, could benefit aphasia-focused drug research, and eventually -if such drugs are proven unequivocally efficient- patients with acquired language disorders.

## Figures and Tables

**Table 1:** An overview of cholinergic pharmacotherapy studies in aphasia.

Study	Study Design and Sample Size	Phase of Stroke–Mean Duration of Aphasia	Lesion Description	Mean Age of Participants	PharmacotHerapy Duration/Dose	Other Type of Treatment	Language Domains	Outcome
	Donepezil							
Pasheka and Bachman 2003 [113]	case study N = 1	Chronic phase 18.5 months post-stroke	not reported	59 years	Donepezil 6 weeks 5 mg	not reported	naming, phrase length, word repetition, Auditory comprehension, attention, motor speech ability	language, cognition and (unexpectedly), motor speech abilities
Berthier et al., 2003 [114]	open-label pilot study N = 10	Chronic phase-4.4 (+/−3.5) years	not reported	56 years	Donepezil 20 weeks 5- and 10- mg	Standard speech and language Therapy two times per week	Phonology discrimination, lexical decision, repetition, short-term memory, naming, lexical knowledge. * *defined by WAB and PALPA*	Improvement in phonemic discrimination, repetition, naming, lexical knowledge.-There were no differences in performance on AQ-WAB and PALPA between 5-mg and 10-mg daily doses.
Berthier et al., 2006 [115]	double-blind, randomized, placebocontrolled, parallel-group study N = 13	chronic aphasia (1 year sinceonset)-33.9 +/− 27.6 months	not reported	48.0 +/− 10.6	Donepezil week 16 5- and 10- mg	Standard speech and language Therapy two times per week	phonemic discrimination, lexical decision, repetition, naming, lexical knowledge * defined by WAB, PALPA and CAL (Aphasia Battery (WAB) and Communicative Activity Log (CAL) (a scale that assesses the patient’s communicative behavior in everyday life)	The severity of aphasia (AQ of the WAB) improved more in the donepezil group than in the placebo group at endpoint. The scores in the picture naming subtest of the PALPA improved more with donepezil at endpoint.
Chen et al., 2010 [116]	a pilot case control study N = 60	Acute phase	not reported	-	Donepezil 12 weeks 5 mg	-	spontaneous speech, comprehension, repetition, and naming * based on WAB *sentence repetition-working memory*	significant recovery in spontaneous speech, comprehension, repetition, and naming
Berthier et al., 2014 [117]	case-series study N = 3	Chronic phase-(>1 year post-stroke)	large left frontotemporo- parietal infarction	58 years	Donepezil 20-week open-label pilot trial and 8-week extension phase No dose reported	Distributed and massed aphasia therapies	sentence repetition-working memory	Combination of donepezil with speech and language therapy provided better results in connected speech during picture description and word list repetition than donepezil and less-intensive therapy.
Yoon, et al., 2015 [118]	Case report N = 1	Chronic phase-8 years	Left temporoparietal and right temporal area	53 years	Donepezil 12 weeks 5 mg/d for 6 weeks and 10 mg/d for the following 6 weeks	No	spontaneous speech, comprehension, repetition, and naming * based on Korean WAB	improvement in comprehension during a conversation and a slight increase of spontaneous speech.
Woodhead et al., 2017 [119]	randomised trial N = 20	3.3 (0.6–8.6) years	average lesion volume = 127.3 (24.2–403.6) cm3)	62.4 (43–90) years	Donepezil 25 weeks 5 mg for the first 5-week block and 10 mg for the second block (*if first block was tolerated*)	Auditory/phonological training (*using Earobics* *software*)	speech comprehension, written comprehension, speech repetition, naming, reading and writing * based on Comprehensive Aphasia Test (CAT)	significant improvement in speech comprehension after phonological training, but worse comprehension on drug than placebo. Both effects were stronger in more severely impaired patients.
Berthier et al., 2017 [120]	Case report N = 1	16 months post-stroke	right striatal-capsular hemorrhage	46-year-old	Donepezil 5 days/week for 12 weeks (total training: 60 h) 5 and 10 mg	audiovisual repetition-imitation therapy (Look-Listen- Repeat-LLR)	Aphasia Severity, daily communication, connected speech production, words and sentences’ repetition, reading and writing.	Treatment with donepezil alone and combined with LLR therapy induced marked improvement in aphasia and communication deficits as well as in selected measures of connected speech production, and phrase repetition. Structural plasticity in the right frontal aslant tract and direct segment of the arcuate fasciculus with both interventions
Berthier et al., 2021 [36]	Case study N = 1	20 months post-stroke	large left fronto-temporo-parietal lesion due to a middle cerebral artery infarction	34-year-old	Donepezil 16 weeks 5 and 10 mg	conventional speech-language therapy (SLT)	Fluency, Comprehension, repetition, naming, comminicativee activity, spoken word-picture matching, semantic paraphasias * defined by WAB	Significant improvement in Naming, Communicative Activity, spoken word-picture matching, non-words repetition, reduction in semantic paraphasias.
Hong et al., 2012 [112]	Galantamine N = 45	Chronic phase-(at least one-year post-onset)/2.2 (1.5 years).	cortical (superficial territory of the middle cerebral artery without involvement of the subcortical grey matter) or subcortical (deep territory of the middle cerebral artery without involvement of the cerebral cortex).	59.1 (+/− 11.4)	Galantamine 16 weeks 8 mg/day increments over 4 weeks up to 16 mg/day	-	spontaneous speech, comprehension, repetition, naming * defined by WAB	Significant improvement in spontaneous speech, comprehension and naming. Subcortical lesion pattern and baseline cognitive function associated with galantamine responsiveness

## Data Availability

The study did not report any data.
